# Kernel Mixture Correntropy Conjugate Gradient Algorithm for Time Series Prediction

**DOI:** 10.3390/e21080785

**Published:** 2019-08-11

**Authors:** Nan Xue, Xiong Luo, Yang Gao, Weiping Wang, Long Wang, Chao Huang, Wenbing Zhao

**Affiliations:** 1 School of Computer and Communication Engineering, University of Science and Technology Beijing, Beijing 100083, China; 2 Institute of Artificial Intelligence, University of Science and Technology Beijing, Beijing 100083, China; 3 Beijing Key Laboratory of Knowledge Engineering for Materials Science, Beijing 100083, China; 4 China Information Technology Security Evaluation Center, Beijing 100085, China; 5 Department of Electrical Engineering and Computer Science, Cleveland State University, Cleveland, OH 44115, USA

**Keywords:** kernel adaptive filtering, conjugate gradient, correntropy, sparsification criterion, malware prediction

## Abstract

Kernel adaptive filtering (KAF) is an effective nonlinear learning algorithm, which has been widely used in time series prediction. The traditional KAF is based on the stochastic gradient descent (SGD) method, which has slow convergence speed and low filtering accuracy. Hence, a kernel conjugate gradient (KCG) algorithm has been proposed with low computational complexity, while achieving comparable performance to some KAF algorithms, e.g., the kernel recursive least squares (KRLS). However, the robust learning performance is unsatisfactory, when using KCG. Meanwhile, correntropy as a local similarity measure defined in kernel space, can address large outliers in robust signal processing. On the basis of correntropy, the mixture correntropy is developed, which uses the mixture of two Gaussian functions as a kernel function to further improve the learning performance. Accordingly, this article proposes a novel KCG algorithm, named the kernel mixture correntropy conjugate gradient (KMCCG), with the help of the mixture correntropy criterion (MCC). The proposed algorithm has less computational complexity and can achieve better performance in non-Gaussian noise environments. To further control the growing radial basis function (RBF) network in this algorithm, we also use a simple sparsification criterion based on the angle between elements in the reproducing kernel Hilbert space (RKHS). The prediction simulation results on a synthetic chaotic time series and a real benchmark dataset show that the proposed algorithm can achieve better computational performance. In addition, the proposed algorithm is also successfully applied to the practical tasks of malware prediction in the field of malware analysis. The results demonstrate that our proposed algorithm not only has a short training time, but also can achieve high prediction accuracy.

## 1. Introduction

Usually, traditional time series prediction methods mainly include autoregression, Kalman filtering, and moving average models. These traditional approaches focus on mathematical statistics and have no capabilities of self-learning, self-organization, and self-adaption. In particular, they cannot be effectively used for data types of nonlinear and multi-feature dimensions in analyzing some complex problems. Currently, there are some machine learning methods developed to address this issue, such as support vector machine (SVM), artificial neural network (ANN), and deep neural network (DNN), then some satisfactory results are achieved in dealing with practical applications, e.g., malware analysis [1,2]. However, there still exists several issues that need to be addressed in using SVM, ANN, and DNN, such as long training time and difficulty in parameter determination. Therefore, it has become critical to seek a better machine learning model [3].

The kernel method as an effective nonlinear system modeling technique has been widely used in the machine learning community [4,5]. Among the available kernel learning methods, the kernel adaptive filtering (KAF) has become a popular calculation method with good computing performance, which has been successfully employed in signal processing, time series prediction, and many others. The main idea of KAF is to map the input data in the original space to the high-dimensional feature space, that is the reproducing kernel Hilbert space (RKHS) [6]. Then, a linear algorithm in this high-dimensional feature space can be applied to solve the nonlinear problem in the original space. The disadvantage of the feature space is that the computational complexity of algorithm increases exponentially with the dimensionality. However, because the kernel method is implicit, it uses kernel function calculation to replace the inner product calculation in high-dimensional feature space. Thus, the issue that the computational complexity increases rapidly with the dimensionality could be effectively avoided.

With the comprehensive study of nonlinear adaptive filters, more extended KAF algorithms have been developed in recent years. In this case, some popular KAF algorithms include kernel least mean squares (KLMS) [7] and kernel recursive least squares (KRLS) [8]. The algorithm KLMS has been widely used in the field of adaptive signal processing due to its simplicity and efficiency [9]. The algorithm KRLS is designed by recursively solving the least squares (LS) problem. Compared with KLMS, KRLS achieves better filtering accuracy and faster convergence speed at the cost of increased computing and storage. Meanwhile, the conjugate gradient (CG) method is another optimization strategy, and it provides a tradeoff between convergence rate and computational complexity, which can generate a better optimal solution than the stochastic gradient descent (SGD) method [10]. Therefore, the method CG has been successfully applied to KAF, and the kernel conjugate gradient (KCG) algorithm was proposed [11]. The KCG with low computational complexity can achieve the same performance as KRLS.

However, the above algorithms are all deduced and experimented in a Gaussian noise environment. This assumption may not be accurate, since the system noise does not always follow the Gaussian distribution in practical applications. Actually, it is accompanied by some impulse noise with low frequency and large amplitude. The interference of this impulse noise on the system is not negligible. In this case, the KAF algorithm derived on the basis of Gaussian noise environment is sensitive to non-Gaussian noise or outliers. As a robust nonlinear similarity measure in kernel space, correntropy has received more and more attention in the field of machine learning and signal processing [12]. Correntropy can capture higher-order statistics of errors and provide significant performance improvement on filtering precision, particularly in non-Gaussian environments. Therefore, more and more algorithms use correntropy as a cost function to improve the stability of algorithms in a non-Gaussian noise environment [13,14]. Furthermore, on the basis of correntropy, the mixture correntropy was proposed [15]. It uses a mixture of two Gaussian functions as a kernel function to enhance flexibility and improve the performance.

Motivated by the work mentioned above, in order to improve the filtering accuracy, convergence speed, and robustness against impulse noise at the same time, the mixture correntropy criterion (MCC) [11] is applied to KCG method. Then, a novel kernel learning algorithm, called kernel mixture correntropy conjugate gradient (KMCCG), is accordingly proposed in this article. In fact, MCC cannot be directly applied to the CG method due to its nonconvexity [15]. Therefore, in accordance with the conjugate function theory, we use the half-quadratic optimization method to transform the mixture correntropy loss into the mixture correntropy half-quadratic function. Then, the mixture correntropy can be smoothly applied to the CG method. Finally, according to the kernel technique, the algorithm KMCCG is proposed for robust online kernel learning. Moreover, in the weight updating, the KAF embeds a growing memory structure, i.e., a growing radial basis function (RBF) network, which leads to the increase of the memory requirement and computation of KAF. There are many traditional methods to restrain the growth of the network structure, such as the novelty criterion [16], the correlation criterion [17], the approximate linear dependence criterion [8], and the surprise criterion [18]. Since the angle between two elements in the Hilbert space can be expressed by the inner product and the similarity of elements in Hilbert space can be measured by the angle, here we use a sparsification criterion based on the angle among elements. The angle criterion used here not only provides geometric intuition, but also offers a simple structure that can be implemented easily.

With the rapid advancement of Internet technology [19], the issue of network security imposes huge challenges to the Internet. Specifically, the demand for malware analysis has become increasingly urgent, and practitioners and researchers have been making progress in the field of malware prediction and detection [20]. Usually, malware is able to implement intention by calling the existing application programming interface (API) in the system. Therefore, the API calling time series as a software behavior is analyzed to achieve malware prediction and detection [21]. Generally speaking, the obtained API call time series can be used to predict future malicious behavior. Specifically, in this article, the proposed algorithm KMCCG is also used in the practical application of malware prediction.

The main contributions of this article are summarized as follows. (1) On the basis of mixture correntropy, a novel robust algorithm KMCCG is proposed through a comprehensive use of the half-quadratic optimization method, the CG technique, and the kernel trick. KMCCG cannot only improve the learning accuracy, but also maintain robustness to impulse noise. (2) In view of the issue that the algorithm KMCCG will produce a growing RBF network, the sparsification criterion based on the angle is used to control the network structure. (3) For a special time series analysis application in relation to malware prediction, KMCCG is accordingly used to achieve this task, which verifies that our proposed algorithm can achieve higher prediction accuracy with less training time.

The rest of this article is organized as follows. In Section 2, the mixture correntropy, the algorithm KCG, and the sparsification criterion are introduced. In Section 3, the details of our algorithm KMCCG are presented. In Section 4, the simulation on time series prediction and the experiment on the malware prediction task are conducted to verify the effectiveness of the our proposed algorithm. The conclusion is summarized in Section 5.

## 2. Related Work

### 2.1. Mixture Correntropy

Correntropy is a local similarity function, which is defined as the generalized correlation in kernel space. It is closely related to the cross-information potential (CIP) in information theory learning (ITL) [22]. It shows very promising results in nonlinear non-Gaussian signal processing. The main property of correntropy is that it provides an effective mechanism to mitigate the influence of large outliers. Recently, correntropy has been successfully applied in various areas, such as signal processing [23], machine learning [24,25,26], adaptive filtering [27,28,29], and others [30,31,32].

The correntropy is used to represent the similarity between two random variables *X* and *Y*. Let $k_{\sigma }\left(\cdot ,\cdot \right)$ be a Mercer kernel function with a kernel bandwidth of σ. Let E[·] be the mathematical expectation. Then, the correntropy can be defined as:

(1) $$\begin{array}{c} V\left(X,Y\right)=E\left[k_{\sigma }\left(X,Y\right)\right]. \end{array}$$

Generally, the Gaussian kernel is the most widely-used kernel in correntropy, and it is as follows:

(2) $$k_{\sigma }\left(X,Y\right)=G_{\sigma }\left(e\right)=\frac{1}{\sqrt{2\pi }\sigma }\exp\left(-\frac{e^{2}}{2\sigma^{2}}\right),$$

where e=X−Y is the error value.

Here, a nonlinear mapping φ(·) is used by the kernel function to map the input space U to high-dimensional space F, and it satisfies $\langle \varphi \left(x\right),\varphi \left(y\right)\rangle =k_{\sigma }\left(X,Y\right)$. Then, (1) is rewritten as:

(3) V(X,Y)=E[〈φ(X),φ(Y)〉].

Since the joint probability density of data in practical applications is usually unknown, for a finite sample ${\left\{x_{i},y_{i}\right\}}_{i=1}^{N}$, the correntropy can be defined as:

(4) $$\hat{V}\left(X,Y\right)=\frac{1}{N}\sum_{i=1}^{N}k_{\sigma }\left(x_{i}-y_{i}\right).$$

Generally, the kernel bandwidth is one of the key parameters in correntropy. Usually, a small kernel bandwidth makes the algorithm more robust to outliers, but it will lead to slow convergence and poor accuracy. On the other hand, when the kernel bandwidth increases, the robustness will be significantly reduced in the case of abnormal values. In order to achieve better performance, a new similarity measure MCC was proposed [11]. It can achieve faster convergence speed and higher filtering accuracy, while maintaining robustness to outliers. The mixture correntropy uses the mixture of two Gaussian functions as the kernel function, and its definition is as follows:

(5) $$M\left(X,Y\right)=E[\alpha G_{\sigma_{1}}\left(e\right)+\left(1-\alpha \right)G_{\sigma_{2}}\left(e\right)],$$

where 0⩽α⩽1 is the mixture coefficient and $\sigma_{1}$ and $\sigma_{2}$ are the kernel bandwidths of the Gaussian functions $G_{\sigma_{1}}\left(\cdot \right)$ and $G_{\sigma_{2}}\left(\cdot \right)$, respectively. When the mixture coefficient α takes a suitable value, the performance of MCC can be better than that of the original correntropy criterion, so the mixture correntropy is a more flexible measure of similarity.

Typically, the empirical mixture correntropy loss can be expressed as $\hat{L}\left(X,Y\right)$ or $\hat{L}\left(e\right)$, where $X={\left[x_{1},x_{2},\cdots ,x_{N}\right]}^{T}$, $Y={\left[y_{1},y_{2},\cdots ,y_{N}\right]}^{T}$, and $e={\left[e_{1},e_{2},\cdots ,e_{N}\right]}^{T}$. Then, it is defined as follows:

(6) $$\begin{array}{cc} \hat{L}\left(X,Y\right) & =1-\hat{M}\left(X,Y\right)\\ & =1-\frac{1}{N}\sum_{i=1}^{N}\left[\alpha G_{\sigma_{1}}\left(e_{i}\right)+\left(1-\alpha \right)G_{\sigma_{2}}\left(e_{i}\right)\right], \end{array}$$

where $e_{i}=x_{i}-y_{i}$.

Here, the Hessian matrix of $\hat{L}\left(e\right)$ with respect to *e* is:

(7) $$H_{\hat{L}\left(e\right)}\left(e\right)=\left[\frac{\partial^{2}\hat{L}\left(e\right)}{\partial e_{i}\partial e_{j}}\right]=\mathrm{diag}\left[\zeta_{1},\zeta_{2},\cdots ,\zeta_{N}\right],$$

where $\zeta_{i}=\alpha \frac{\sigma_{1}^{2}-e_{i}^{2}}{N\sigma_{1}^{4}}G_{\sigma_{1}}\left(e_{i}\right)+\left(1-\alpha \right)\frac{\sigma_{2}^{2}-e_{i}^{2}}{N\sigma_{2}^{4}}G_{\sigma_{2}}\left(e_{i}\right)$. Here, if $\left|e_{i}\right|⩽\sigma_{1}⩽\sigma_{2}$, then $\zeta_{i}>0$. For any point that satisfies ${∥e∥}_{\infty }⩽\sigma_{1}⩽\sigma_{2}$, there is $H_{\hat{L}\left(e\right)}\left(e\right)⩾0$.

It can be seen that the Hessian matrix $H_{\hat{L}\left(e\right)}$ of empirical mixture correntropy loss, as a function of *e*, is convex when the condition ${∥e∥}_{\infty }=\max_{1⩽i⩽N}\left|e_{i}\right|⩽\sigma_{1}$ is satisfied. Therefore, the use of mixture correntropy as a loss function cannot be directly applied to convex optimization [33].

### 2.2. Kernel Conjugate Gradient Algorithm

The CG is a specific method between the steepest descent and the Newton methods. It accelerates the typically slow convergence associated with the steepest descent, while avoiding the information requirements associated with the evaluation, storage, and inversion of Hessian as required by Newton’s method [10].

Let $A\in R^{n\times n}$ be a symmetric positive definite matrix and $d_{1},d_{2},\cdots ,d_{m}$ be a set of non-zero vectors in $R^{n}$. If $d_{i}^{T}Ad_{j}=0\;\left(i\neq j\right)$, then we think that $d_{1},d_{2},\cdots ,d_{m}$ are conjugated to each other about A.

There is an unconstrained optimization problem as follows:

(8) $$\min_{x\in R^{n}}f\left(x\right),$$

where *f* is a continuous differentiable function on $R^{n}$. The nonlinear method CG for solving the above (8) has the following iterative format:

(9) $$\begin{array}{cc} x_{k+1} & =x_{k}+\alpha_{k}p_{k},\\ p_{k} & =\left\{\begin{array}{cc} -g_{k}, & k=1\\ -g_{k}+\beta_{k}p_{k-1}, & k⩾2 \end{array}\right. \end{array}$$

where $g_{k}=\nabla f\left(x_{k}\right),p_{k}$ is the search direction, $\alpha_{k}>0$ is the step factor, $\beta_{k}$ is a certain parameter, and different $\beta_{k}$ corresponds to a different CG method. That is, when f(x) is a strictly convex quadratic function, the search direction *p* generated by the method (9) is conjugated to the Hessian matrix of f(x). The process of CG iteration is described in Algorithm 1, where $r_{i}$ is the residual vector, $p_{i}$ is the search direction, $x_{i}$ is the approximate solution. In addition, $\alpha_{i}$ and $\beta_{i}$ are the step factors, and the stopping criterion is that the algorithm achieves convergence.

**Algorithm 1** The conjugate gradient (CG) algorithm.**Input:** Given symmetric positive definite matrix A; Given the vector b; Given the initial iteration value x; **Initialization:** $r_{0}=b-Ax_{0}$; $p_{0}=r_{0}$; **repeat**   **for**k=0,1,…**do**      **if**$p_{k}=0$**then**         return $x_{0}$;      **endelse**         $\alpha_{k}=\frac{r_{k}^{T}r_{k}}{p_{k}^{T}Ap_{k}}$;         $x_{k+1}=x_{k}+\alpha_{k}p_{k}$;         $r_{k+1}=r_{k}-\alpha_{k}Ap_{k}$;         $\beta_{k}=\frac{r_{k+1}^{T}r_{k+1}}{r_{k}^{T}r_{k}}$;         $p_{k+1}=r_{k+1}+\beta_{k}p_{k}$;      **end**   **end** **until** Stopping_Criterion is met.

In order to address the nonlinear problem effectively, the KCG algorithm was proposed for online application [11]. In an effort to use kernel techniques, the solution vector of the algorithm CG is represented by a linear combination of input vectors. Then, the convergence speed of the online algorithm KCG is much faster than that of the algorithm KLMS. Actually, the KCG achieves the same convergence performance as the KRLS in many cases; however, the computational cost is greatly reduced [11]. Another attractive feature of KCG is that it does not require the user-defined parameters. The algorithm KCG is described as Algorithm  2. In this algorithm, G is the Gram matrix, η is the coefficient vector, κ(·,·) is the Mercer kernel, *M* is the size of the dictionary, $r_{0}$ and $r_{1}$ are the residual vectors, and e is the error vector. Moreover, $\alpha_{1}$, $\alpha_{2}$, and $\beta_{2}$ are step sizes; $S_{1}$ is the residual vector of the normal equation; $v_{1}$ and $v_{2}$ are intermediate vectors; and H stands for the conjugate transpose.

**Algorithm 2** The kernel conjugate gradient (KCG) algorithm.**Initialization:** $X_{1}=x_{1};\;\;q_{1}=\kappa \left(x_{1},x_{1}\right);\;\;q_{1}=\left[\sqrt{q_{1}}\right]$; $G_{1}=q_{1};\;\;\eta_{1}=\frac{{\bar{d}}_{1}}{q_{1}};\;\;e_{1}=0;\;\;M=1$; **repeat**   **for**k=2,3,…**do**      $q_{k}=\kappa \left(x_{k},x_{k}\right)$;      $g_{k}=\left[\kappa \left(X_{M}\left(:,1\right),x_{k}\right),\ldots ,\kappa \left(X_{M}\left(:,M\right),x_{k}\right)\right]$;      $\nu_{m}=\frac{g_{k}\left(m\right)}{\sqrt{q_{k}}q_{k-1}\left(m\right)};\;\left(m=1,2,\ldots ,M\right)$      **if**$\max\left\{\left|\nu_{m}\right|\right\}<\nu_{0}$**then**         M=M+1;         $X_{M}=\left[X_{M-1},x_{k}\right];\;\;q_{M}=\left[q_{M-1},\sqrt{q_{k}}\right]$;         $G_{M}=\left[\begin{array}{cc} G_{M-1} & g_{k}^{T}\\ {\bar{g}}_{k} & q_{k} \end{array}\right]$;         $r_{0}={\left[e_{M-1},d_{k}-g_{k}{\bar{\eta }}_{M-1}\right]}^{H};\;\;v_{1}=G_{M}r_{0}$;         $\gamma_{0}=\langle v_{1},r_{0}\rangle ;\;\;\alpha_{1}=\frac{\gamma_{0}}{\langle v_{1},v_{1}\rangle }$;         $r_{1}=r_{0}-\alpha_{1}v_{1};\;\;s_{1}=G_{M}r_{1}$;         $\gamma_{1}=\langle s_{1},r_{1}\rangle ;\;\;\beta_{2}=\frac{\gamma_{1}}{\gamma_{0}}$;         $v_{2}=\beta_{2}v_{1}+s_{1};\;\;\alpha_{2}=\frac{\gamma_{1}}{\langle v_{2},v_{2}\rangle }$;         $\eta_{M}=\left[\eta_{M-1},0\right]+\left(\alpha_{1}+\alpha_{2}\beta_{2}\right)r_{0}+\alpha_{2}r_{1}$;         $e_{M}={\left(r_{1}-\alpha_{2}v_{2}\right)}^{H}$;      **end**   **end** **until** Stopping_Criterion is met.

Since the algorithm KCG is derived from the solution of the least squares problem, good performance can be maintained in a Gaussian noise environment [11]. However, in the non-Gaussian case, the performance of KCG may not be satisfactory [11]. Therefore, we used the mixture correntropy as the loss function and propose the algorithm KMCCG.

### 2.3. Sparsification Criterion

KAF uses an online approaching strategy, that is each time a new set of data arrives, it is allocated a storage unit. The linear growth of the network structure leads to an increase in the memory requirements and calculations of KAF. Since the angle between two elements in the feature space can be represented by the inner product and the similarity of the elements in the space can be measured by an angle, then a simple sparsification criterion on the basis of the angle between elements in RKHS is used to control the network structure [11]. The cosine of the angle between φ(x) and φ(y) is as follows:

(10) $$\nu \left(x,y\right)=\frac{\langle \varphi (x),\varphi (y)\rangle }{∥\varphi (x)∥\cdot ∥\varphi (y)∥}=\frac{\kappa (y,x)}{\sqrt{\kappa (x,x)\kappa (y,y)}}.$$

The algorithm reconstructs the network “dictionary” through the sparsification criterion. If the current dictionary is *D* and a new sample $\left(x_{k},d_{k}\right)$ is coming, the procedure of the angle criterion-based sparsification can be described through the following two steps.

First, the parameter *v* is calculated:

(11) $$\nu_{k}=\max_{1⩽m⩽M}\left|\nu \left(x_{k},{\tilde{x}}_{m}\right)\right|\in \left[0,1\right].$$

Second, if $\nu_{k}$ is smaller than the predefined threshold $\nu_{0}$, $\left(\varphi \left(x_{k}\right),d_{k}\right)$ is added to *D*, otherwise it is discarded. Because the parameter $\nu_{0}$ represents the level of similarity between the new element and those old elements in *D*, we call it the similarity parameter.

## 3. The Proposed Algorithm

### 3.1. Half-Quadratic Optimization of the Mixture Correntropy

For the mixture correntropy loss function (5), since its Hessian matrix is not positive definite, its global convexity cannot be guaranteed. Therefore, the mixture correntropy loss cannot be directly applied to the convex optimization problem. Fortunately, the half-quadratic (HQ) optimization method is an effective method to address the non-convex optimization problem [33], which converts the original objective function into the half-quadratic objective function. In this article, the HQ optimization method is used to transform the mixture correntropy loss function, and then, the method CG is employed to solve the transformation function.

Since the mixture correntropy is the sum of two exponential functions, we let f(x)=exp(−x) be an exponential function. The conjugate function of f(x) is g(v)=−vln(−v)+v(v<0). According to the theory of the conjugate function, the conjugate function of g(v) is given by:

(12) $$g^{*}\left(u\right)=\underset{v<0}{\sup}\left\{uv-g\left(v\right)\right\}=\underset{v<0}{\sup}\left\{uv+v\ln\left(-v\right)-v\right\}.$$

Let f(v)=uv+vln(−v)−v, where v<0. Then, f(v) reaches the maximum value exp(−u) at v=−exp(−u). Therefore, we can get:

(13) $$g^{*}\left(u\right)=\underset{v<0}{\sup}\left\{uv+v\ln\left(-v\right)-v\right\}=\exp\left(-u\right),$$

where v=−exp(−u).

When $u=\frac{e_{k}^{2}}{2\sigma^{2}}$, we can get:

(14) $$g^{*}\left(\frac{e_{k}^{2}}{2\sigma^{2}}\right)=\underset{v<0}{\sup}\left\{v\frac{e_{k}^{2}}{2\sigma^{2}}+v\ln\left(-v\right)-v\right\}=\exp\left(-\frac{e_{k}^{2}}{2\sigma^{2}}\right),$$

where $v=-\exp\left(-\frac{e_{k}^{2}}{2\sigma^{2}}\right)$.

Then, (5) can be written as:

(15) $$\begin{array}{cc} M(X,Y) & =\frac{\alpha }{N}\sum_{i=1}^{N}\kappa_{\sigma_{1}}\left(x_{i},y_{i}\right)+\frac{1-\alpha }{N}\sum_{i=1}^{N}\kappa_{\sigma_{2}}\left(x_{i},y_{i}\right)\\ & =\frac{\alpha }{N}\sum_{i=1}^{N}\exp\left(-\frac{e_{i}^{2}}{2\sigma_{1}^{2}}\right)+\frac{1-\alpha }{N}\sum_{i=1}^{N}\exp\left(-\frac{e_{i}^{2}}{2\sigma_{2}^{2}}\right)\\ & =\frac{\alpha }{N}\underset{v<0}{\sup}\left\{v_{i}\frac{e_{k}^{2}}{2\sigma_{1}^{2}}-g\left(v_{i}\right)\right\}+\frac{1-\alpha }{N}\underset{v^{\prime }<0}{\sup}\left\{v_{i}^{\prime }\frac{e_{k}^{2}}{2\sigma_{2}^{2}}-g\left(v_{i}^{\prime }\right)\right\}. \end{array}$$

Because the solution to the mixture correntropy Loss (6) is equivalent to solving the following problem:

(16) $$\begin{array}{cc} \max_{v<0}M\left(X,Y\right) & =\frac{\alpha }{N}\max_{v<0}\left\{v_{i}\frac{e_{k}^{2}}{2\sigma_{1}^{2}}-g\left(v_{i}\right)\right\}+\frac{1-\alpha }{N}\max_{v^{\prime }<0}\left\{v_{i}^{\prime }\frac{e_{k}^{2}}{2\sigma_{2}^{2}}-g\left(v_{i}^{\prime }\right)\right\}, \end{array}$$

therefore, by solving the sum of the following weighted least squares problems, the equivalent solution of the mixture correntropy can be obtained.

(17) $$\frac{\alpha }{N}\min\sum_{i=1}^{N}\left(-v_{i}\frac{e_{i}^{2}}{2\sigma_{1}^{2}}\right)+\frac{1-\alpha }{N}\min\sum_{i=1}^{N}\left(v_{i}^{\prime }-\frac{e_{i}^{2}}{2\sigma_{2}^{2}}\right),$$

where $v_{i}=-\exp\left(-\frac{e_{i}^{2}}{2\sigma_{1}^{2}}\right)$, $v_{i}^{\prime }=-\exp\left(-\frac{e_{i}^{2}}{2\sigma_{2}^{2}}\right)$, $e_{i}=y_{i}-f\left(x_{i}\right)$.

The Hessian matrix of the weighted least squares problem (17) is as follows:

(18) $$H\left(e\right)=\mathrm{diag}\left[-\frac{\alpha v_{1}}{\sigma_{1}^{2}},-\frac{\alpha v_{2}}{\sigma_{1}^{2}},\ldots ,-\frac{\alpha v_{N}}{\sigma_{1}^{2}}\right]+\mathrm{diag}\left[-\frac{\left(1-\alpha \right)v_{1}^{\prime }}{\sigma_{2}^{2}},-\frac{\left(1-\alpha \right)v_{2}^{\prime }}{\sigma_{2}^{2}},\ldots ,-\frac{\left(1-\alpha \right)v_{N}^{\prime }}{\sigma_{2}^{2}}\right],$$

where $v_{i}<0$ and $v_{i}^{\prime }<0$. Hence, we obtain H(e)>0, and then, (17) is a global convex optimization problem. Here, when the Hessian matrix is positive definite, it means that the function is a convex function. Then, the objective function can be optimized by the conjugated gradient method.

### 3.2. Kernel Mixture Correntropy Conjugate Gradient Algorithm

The core of KAF is to transform the input data into a high-dimensional feature space through the kernel function. The inner product operation in the feature space is more efficiently calculated by the kernel technique. Its goal is to get the mapping function f(x) between the input and output. According to the adaptive filtering theory, we can obtain:

(19) $$f\left(x_{k}\right)=\sum_{i=1}^{k}\eta_{i}\kappa \left(\cdot ,x_{k}\right),$$

where $\eta_{i}$ is the expansion coefficient and $\kappa \left(\cdot ,x_{k}\right)$ is a kernel function with the center $x_{k}$.

According to (17), we consider the following kernel-induced weighted least squares problem:

(20) $$\begin{array}{c} \begin{array}{cc} & \frac{\alpha }{N}\min\sum_{i=1}^{k}\left(-\frac{v_{i}}{2\sigma_{1}^{2}}{\left(d_{i}-\eta_{i}\kappa \left(\cdot ,x_{k}\right)\right)}^{2}\right)+\frac{1-\alpha }{N}\min\sum_{i=1}^{k}\left(-\frac{v_{i}^{\prime }}{2\sigma_{2}^{2}}{\left(d_{i}-\eta_{i}\kappa \left(\cdot ,x_{k}\right)\right)}^{2}\right)\\ = & \frac{\alpha }{N}\min{∥\sqrt{V}\left(d^{T}-G_{1}\eta \right)∥}^{2}+\frac{1-\alpha }{N}\min{∥\sqrt{V^{\prime }}\left(d^{T}-G_{2}\eta \right)∥}^{2}, \end{array} \end{array}$$

where $\eta ={\left[\eta_{1},\eta_{2},\ldots ,\eta_{k}\right]}^{T}$ is the expansion coefficient and G is the Gram matrix, which is defined as:

(21) $$G_{k}=\left[\begin{array}{cccc} \kappa \left(x_{1},x_{1}\right) & \kappa \left(x_{1},x_{2}\right) & \cdots & \kappa \left(x_{1},x_{k}\right)\\ \kappa \left(x_{2},x_{1}\right) & \kappa \left(x_{2},x_{2}\right) & \cdots & \kappa \left(x_{2},x_{k}\right)\\ \vdots & \vdots & \ddots & \vdots \\ \kappa \left(x_{k},x_{1}\right) & \kappa \left(x_{k},x_{2}\right) & \cdots & \kappa \left(x_{k},x_{k}\right) \end{array}\right].$$

Then, we use the method CG to solve the weighted least squares problem (20). The major work of the online algorithm KMCCG lies in the update of the Gram matrix $G_{M}$ and the coefficient vector $\eta_{M}$. The Gram matrix $G_{M}$ can be updated as follows:

(22) $$G_{M}=\left[\begin{array}{cc} G_{M-1} & g_{M}^{T}\\ {\bar{g}}_{M} & q_{M} \end{array}\right],$$

where $q_{M}=\kappa \left(x_{M},x_{M}\right)$ and $g_{M}=\left[\kappa \left(x_{1},x_{M}\right),\kappa \left(x_{2},x_{M}\right),\ldots ,\kappa \left(x_{M-1},x_{M}\right)\right]$. Because ${\left[\eta_{M-1},0\right]}^{T}$ is a good approximation of $\eta_{M}$, only a few iterations (one or two) with this initial vector can achieve satisfactory performance. We use ${\left[\eta_{M-1},0\right]}^{T}$ as the initial value of $\eta_{M}$, and the initial residual $r_{0}$ can be expressed as follows:

(23) $$\begin{array}{cc} r_{0} & =\alpha V_{k}\left(d^{T}-G_{k}{\left[\eta_{M-1},0\right]}^{T}\right)+\left(1-\alpha \right)V_{k}^{\prime }\left(d^{T}-G_{k}{\left[\eta_{M-1},0\right]}^{T}\right)\\ & =\alpha V_{k}\left(\left[\begin{array}{c} d_{k-1}^{T}\\ d_{k} \end{array}\right]-\left[\begin{array}{cc} G_{k-1} & g_{k}^{T}\\ {\bar{g}}_{k} & q_{k} \end{array}\right]\left[\begin{array}{c} \eta_{k-1}\\ 0 \end{array}\right]\right)+\left(1-\alpha \right)V_{k}^{\prime }\left(\left[\begin{array}{c} d_{k-1}^{T}\\ d_{k} \end{array}\right]-\left[\begin{array}{cc} G_{k-1} & g_{k}^{T}\\ {\bar{g}}_{k} & q_{k} \end{array}\right]\left[\begin{array}{c} \eta_{k-1}\\ 0 \end{array}\right]\right)\\ & =\alpha {\left[e_{k-1},v_{k}\left(d_{k}-{\bar{g}}_{k}\eta_{k-1}\right)\right]}^{T}+\left(1-\alpha \right){\left[e_{k-1},v_{k}^{\prime }\left(d_{k}-{\bar{g}}_{k}\eta_{k-1}\right)\right]}^{T}\\ & =\alpha {\left[e_{k-1},v_{k}e_{k}\right]}^{T}+\left(1-\alpha \right){\left[e_{k-1},v_{k}^{\prime }e_{k}\right]}^{T}, \end{array}$$

where $V_{k}=\left[\begin{array}{cc} I & 0\\ 0^{T} & v_{k} \end{array}\right]$, $V_{k}^{\prime }=\left[\begin{array}{cc} I & 0\\ 0^{T} & v_{k}^{\prime } \end{array}\right]$, $v_{k}=\frac{1}{\sigma^{2}}\exp\left(-\frac{e_{k}^{2}}{2\sigma_{1}^{2}}\right)$, and $v_{k}^{\prime }=\frac{1}{\sigma^{2}}\exp\left(-\frac{e_{k}^{2}}{2\sigma_{2}^{2}}\right)$. Then, we can get the KMCCG, as shown in Algorithm 3.

What follows is a working example we provide for this section to show the evolution of values throughout the whole process. Let $x_{1}=\left[0,0,0,0,1.0399\right]$, $d_{1}=\left[1.5700\right]$, $x_{2}=\left[0,0,0,1.0399,1.5700\right]$, and $d_{2}=\left[1.3156\right]$. Then, according to Algorithm 3, the evolution of the values is as follows.

When k=2, $q_{2}=\kappa \left(x_{2},x_{2}\right)=0.6595$, $g_{2}=\kappa \left(x_{1},x_{2}\right)=0.3337$, and $v_{1}=0.3337$, then because $\max\left\{\left|v_{1}\right|\right\}<v_{0}=0.8$, $x_{2}$ is added to the dictionary, which means that M=M+1=2 and $X_{2}=\left[X_{1},x_{2}\right]$. The Gram matrix $G_{M}$ can be updated as follows:

(24) $$G_{2}=\left[\begin{array}{cc} G_{1} & g_{2}^{T}\\ {\bar{g}}_{2} & q_{2} \end{array}\right].$$

Then, the residual can be obtained as $r_{0}={\left[0,0.3991\right]}^{T}$ and $r_{1}={\left[-0.1834,0.1210\right]}^{T}$. On the basis of this, the correlation coefficient $\eta_{2}$ can be updated. When the new input arrives, the algorithm will continuously update the correlation coefficient to make it more suitable for the next input. Finally, the algorithm will stop when the convergence condition is satisfied, that is it reaches the maximum number of iterations.

**Algorithm 3** The kernel mixture correntropy conjugate gradient (KMCCG) algorithm.**Initialization:** $X_{1}=x_{1};\;\;q_{1}=\kappa \left(x_{1},x_{1}\right);\;\;q_{1}=\left[\sqrt{q_{1}}\right]$; $G_{1}=q_{1};\;\;\eta_{1}=\frac{{\bar{d}}_{1}}{q_{1}};\;\;e_{1}=0;\;\;M=1$; **repeat**   **for**k=2,3,…**do**      $q_{k}=\kappa \left(x_{k},x_{k}\right)$;      $g_{k}=\left[\kappa \left(X_{M}\left(:,1\right),x_{k}\right),\ldots ,\kappa \left(X_{M}\left(:,M\right),x_{k}\right)\right]$;      $\nu_{m}=\frac{g_{k}\left(m\right)}{\left[\sqrt{q_{k}}q_{k-1}\left(m\right)\right]};\;\;\left(m=1,2,\ldots ,M\right)$      **if**$\max\left\{\left|\nu_{m}\right|\right\}<\nu_{0}$**then**         M=M+1;         $X_{M}=\left[X_{M-1},x_{k}\right];\;\;q_{M}=\left[q_{M-1},\sqrt{q_{k}}\right]$;         $G_{M}=\left[\begin{array}{cc} G_{M-1} & g_{k}^{T}\\ {\bar{g}}_{k} & q_{k} \end{array}\right]$;         $e_{k}=d_{k}-{\bar{g}}_{k}\eta_{k-1}$;         $v_{k}=\frac{1}{\sigma_{1}^{2}}\cdot \exp\left(-\frac{e_{k}^{2}}{2\sigma_{1}^{2}}\right);\;\;v_{k}^{\prime }=\frac{1}{\sigma_{2}^{2}}\cdot \exp\left(-\frac{e_{k}^{2}}{2\sigma_{2}^{2}}\right)$;         $r_{0}=\alpha {\left[e_{k-1},v_{k}e_{k}\right]}^{T}+\left(1-\alpha \right){\left[e_{k-1},v_{k}^{\prime }e_{k}\right]}^{T};\;\;v_{1}=G_{M}r_{0}$;         $\gamma_{0}=\langle v_{1},r_{0}\rangle ;\;\;\alpha_{1}=\frac{\gamma_{0}}{\langle v_{1},v_{1}\rangle }$;         $r_{1}=r_{0}-\alpha_{1}v_{1};\;\;s_{1}=G_{M}r_{1}$;         $\gamma_{1}=\langle s_{1},r_{1}\rangle ;\;\;\beta_{2}=\frac{\gamma_{1}}{\gamma_{0}}$;         $v_{2}=\beta_{2}v_{1}+s_{1};\;\;\alpha_{2}=\frac{\gamma_{1}}{\langle v_{2},v_{2}\rangle }$;         $\eta_{M}=\left[\eta_{M-1},0\right]+\left(\alpha_{1}+\alpha_{2}\beta_{2}\right)r_{0}+\alpha_{2}r_{1}$;         $e_{M}={\left(r_{1}-\alpha_{2}v_{2}\right)}^{H}$;      **end**   **end** **until** Stopping_Criterion is met.

### 3.3. Computational Time Complexity Analysis

As shown in Table 1, we obtain the computational time complexity of four algorithms KLMS, KRLS, KCG, and KMCCG, after analyzing the implementation process of those algorithms. In this table, M is the dictionary size in the algorithm. KLMS is a simple KAF with minimal computational complexity. KRLS requires $\left(4M^{2}+4M\right)$ additions, $\left(4M^{2}+4M\right)$ multiplications, and one division per iteration, while KCG achieves a convergence speed and filtering accuracy comparable to KRLS [11], and its computational complexity is relatively small. In addition, compared with KCG, KMCCG requires two divisions and one multiplication when calculating $v_{k}$ and also requires an addition and a multiplication to update the residual vector $r_{0}$. Table 1 shows that in each iteration, KMCCG requires fewer additions and multiplications than KRLS, but requires four more division operations. Because the number of instruction cycles required by the division operation is generally 20-times that of the addition operation and M is usually greater than 100, the computational complexity of KMCCG is still lower than that of the KRLS algorithm. Moreover, when the input vector does not meet the sparsification criterion, there is no additional calculation for KMCCG, but there are still $\left(4M^{2}+4M\right)$ additions and $\left(4M^{2}+4M\right)$ multiplications for KRLS. Therefore, KMCCG can achieve higher prediction accuracy with less computational and storage costs.

## 4. Experimental Results and Discussions

In this section, the experiments on short-term predictions of the Mackey–Glass chaotic time series, minimum daily temperatures time series, and the real-world malware API call sequence are conducted to illustrate the performance of our proposed algorithm.

### 4.1. Mackey–Glass Time Series Prediction

The chaotic time series is one of the fundamental forms of movement in nature and human society. Generally, the classical Mackey–Glass chaotic time series is generated by the following differential equation [6]:

(25) $$\frac{dx(t)}{dt}=-bx\left(t\right)+\frac{ax(t-\tau )}{1+x{\left(t-\tau \right)}^{n}},$$

where the parameters are set to a=0.2,b=0.1,n=10, and τ=30. Moreover, the sampling period is 6 s. This experiment uses the past seven samples $u\left(k\right)=\left[x(k),x(k-1),\ldots ,x(k-6)\right]$ to predict the current input d(k)=x(k+1). Then, we use the dimensions of the matrix to represent the size of the matrix. Therefore, the size of the training input set is (7×1000), the size of the training target set is (1000×1), the size of the testing input set is (7×200), and the size of the testing target set is (200×1).

The algorithm KMCCG was compared with the quantized KLMS (QKLMS) algorithm [34], the quantized kernel maximum correntropy (QKMC) algorithm [35], and the kernel maximum mixture correntropy (KMMCC) algorithm [15] in four different noise environments, to verify the performance of our proposed algorithm. Here, QKLMS is one of the most classical KAF algorithms based on the mean square error (MSE) criterion, and it achieves good prediction accuracy in the Gaussian noise environment. QKMC is a KAF algorithm based on correntropy, which can also achieve good prediction accuracy and maintain robustness. The recently-proposed algorithm KMMCC combined with the mixture correntropy criterion has been demonstrated to be able to obtain satisfactory prediction accuracy and robustness. All algorithms were configured with a Gaussian kernel. For a fair comparison, the optimal parameter setting was conducted to let each algorithm achieve the desirable performance. Finally, the performance of the algorithm was evaluated by MSE, which is defined here as follows:

(26) $$\mathrm{MSE}=10\log_{10}\left(\frac{1}{N}\sum_{i=1}^{N}{\left(d\left(i\right)-y\left(i\right)\right)}^{2}\right),$$

where *N* represents the number of predicted values.

**Figure 1:**
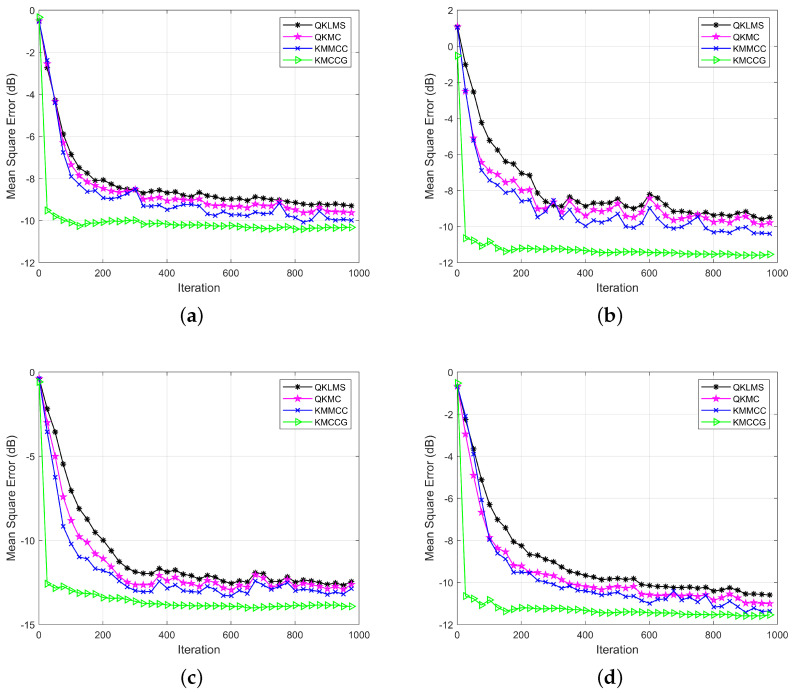
Mackey–Glass time series prediction: learning performance in terms of the testing MSE (mean square error) for QKLMS (quantized kernel least mean squares), QKMC (quantized kernel maximum correntropy), KMMCC (kernel maximum mixture correntropy), and KMCCG (kernel mixture correntropy conjugate gradient) under different noise environments: (**a**) Gaussian; (**b**) Bernoulli; (**c**) sine wave; (**d**) uniform.

Figure 1 shows the learning performance of these algorithms in four noise environments. Obviously, in all four types of noise environments, the testing MSE of KMCCG was smaller than that of the stochastic gradient-based filtering algorithms, i.e., QKLMS, QKMC, and KMMCC. Meanwhile, the convergence speed of KMCCG was obviously faster than that of the other compared algorithms. This verifies that the CG technique used in KMCCG can achieve a faster convergence speed and higher learning accuracy. Therefore, the algorithm KMCCG can achieve the best performance in all the compared algorithms.

### 4.2. Minimum Daily Temperatures Time Series Prediction

In this section, the minimum daily temperatures time series is selected as the dataset to verify the performance of the proposed algorithm. This dataset describes the minimum daily temperature in Melbourne, Australia, for 10 years (1981–1990) [36]. The unit is Celsius, and there are 3650 observations. The data source is the Australian Meteorological Agency.

Here, we use the previous five input samples $x\left(k\right)={\left[x\left(k-5\right),x\left(k-4\right),\ldots ,x\left(k-1\right)\right]}^{T}$ to predict the current point d(k)=x(k), where x(k) and d(k) represent the input vector and the corresponding expected output, respectively. Additionally, the size of the training input set as (5×1000); the size of the training target set was (1000×1); the size of the test input set was (5×200); and the size of the test target set was (200×1). Finally, the MSE was also used to evaluate the performance of those algorithms. Then, our algorithms was compared with QKLMS, QKMC, and KMMCC to verify the computational performance.

**Figure 2:**
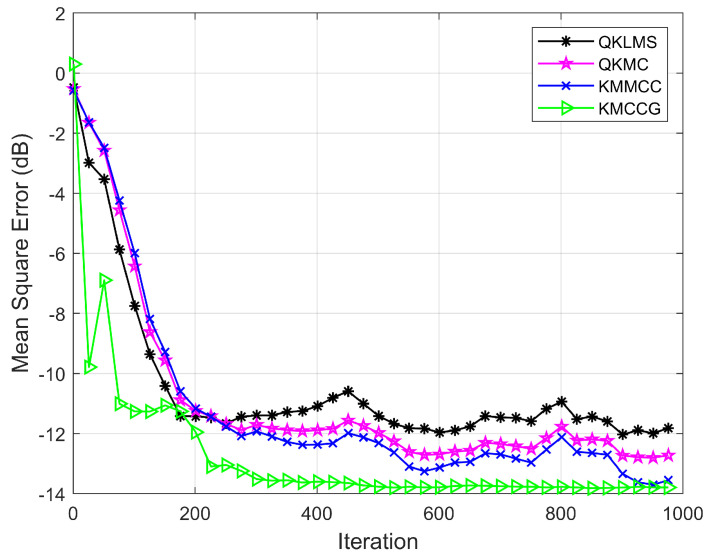
Minimum daily temperatures time series prediction: learning performance in terms of the testing MSE for QKLMS, QKMC, KMMCC, and KMCCG.

Figure 2 shows the learning curve for these algorithms. Obviously, the testing MSE of KMCCG is less than that of QKLMS, QKMC, and KMMCC, which demonstrates that the proposed algorithm can perform better than all three other algorithms.

### 4.3. Malware API Call Sequence Prediction

In this section, We apply the proposed algorithm to the malware API call sequence prediction, while verifying the effectiveness of our algorithm through the actual time series data. The purpose of this experiment is to predict what the next API would be, which can be used to determine whether it is malware or not.

#### 4.3.1. Background

API is the service interface provided by the operating system. Applications call the API when completing file reading and writing, network access, and other tasks [37]. Meanwhile, malware also needs to call the API when implementing functions. Hence, it is an effective method to predict and detect malware behavior by extracting the API call sequence [21].

With the rapid advances in computational intelligence methodology, using machine learning algorithms to predict malware via the API call sequence can make the malware prediction more intelligent, and the new malware can be detected in a more timely manner [38]. In this field, SVM, ANN, and other methods have been applied to malware prediction and detection, and some satisfactory results are achieved.

In [39], with the help of global features using the Gabor wavelet transform and Gist, the feed-forward ANN was developed to identify the behavior of malicious data with a good accuracy. In [40], after abstracting the complex behaviors based on the semantic analysis of dynamic API sequences, an SVM was proposed to achieve malware detection with good generalization ability. Furthermore, with the popular use of the deep learning method, some DNN models were also applied to tackle the issue of malware detection. For instance, in [41], the features were extracted from five minutes of API call log sequences by using a recurrent neural network, and then, they were input to the convolutional neural network to achieve deep learning with the purpose of malware detection.

Although some good performances have been achieved by using the above approaches, there still exist several limitations, such as the long training time and difficulty in parameter determination. Since mixture correntropy as a new measure of local similarity defined in kernel space can be used to address large outliers, hence, in order to reduce the training time while maintaining high prediction accuracy and robustness to abnormal data in the API call sequence, our algorithm KMCCG can be considered to cope with the malware prediction. Here, it should be noted that although some traditional machine learning-based malware prediction and detection algorithms may be vulnerable to adversarial methods or tools, such as EvadeML [42] and poisoning attack [43], the algorithm KMCCG may be a better choice in malware prediction, in consideration of the satisfactory robustness achieved by using mixture correntropy.

We mainly analyzed the acquired API call sequence and predicted the malicious behavior that may occur in the future using our proposed kernel learning algorithm. Then, through the combination of these predicted malicious behaviors with the actual detected malicious behaviors, we will extract feature vectors and integrate them as the discriminant basis of malware detection. In so doing, we can determine whether the application belongs to malware or not, through the machine learning classification model.

#### 4.3.2. Experimental Result

API call information can be extracted by static and dynamic methods. Through the use of the static method [44], the API list can be extracted from the portable executable (PE) format of the executable files. Furthermore, with the dynamic method [45], the called API can be observed by running the executable files.

**Figure 3:**
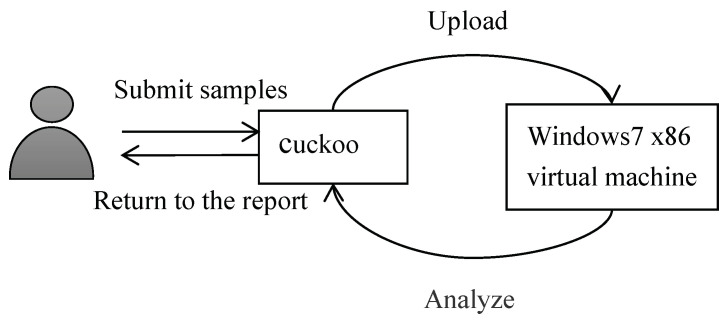
Flowchart for building a malware corpus.

**Figure 4:**
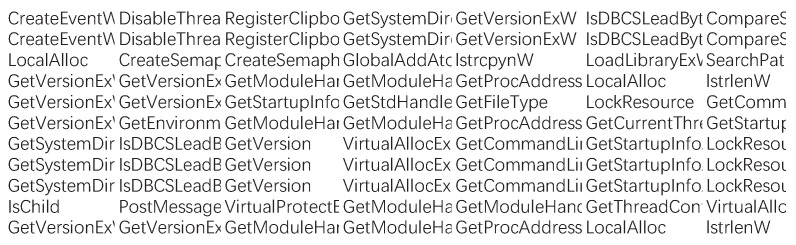
Malware API (application programming interface) call time series report.

While creating the dataset, we randomly selected Windows malware samples from the malware datasets of Dasmalwerk and VirusShare and put the software into the cuckoo sandbox to analyze the report automatically. In order to avoid related security issues caused by malware propagation and accidental execution, here we chose to deploy the cuckoo sandbox to the Ubuntu environment. Figure 3 shows a flowchart for building the malware corpus. The specific analysis process of the sample is as follows: (1) The first step is to launch cuckoo on the Linux platform. (2) Then, we submit the sample to be analyzed to cuckoo. (3) Cuckoo uploads the sample to the virtual machine and collects the behavior data. (4) After the analysis is completed, cuckoo will generate an analysis report in its own working directory. Then, the API sequence is extracted from the report, and Figure 4 shows some API call time series. Each line in this figure represents a malware API call time series. In the following experiment, the API call sequence of a certain malware sample is selected as the dataset.

**Figure 5:**
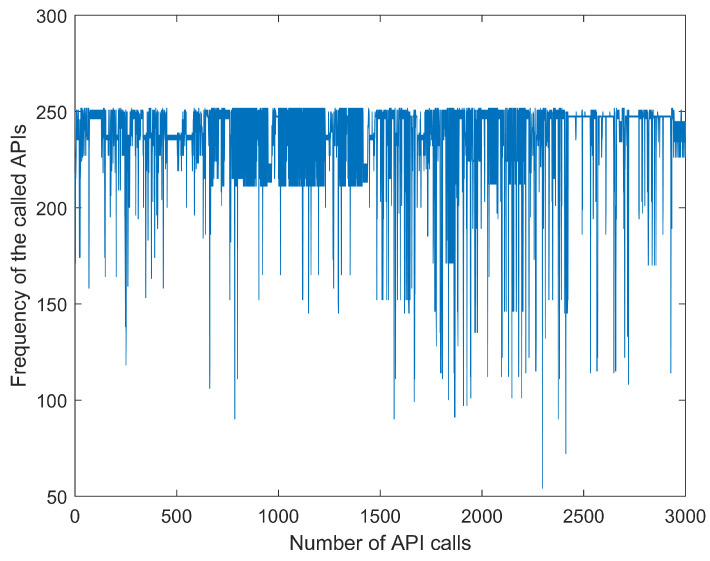
Original malware API call time series.

**Figure 6:**
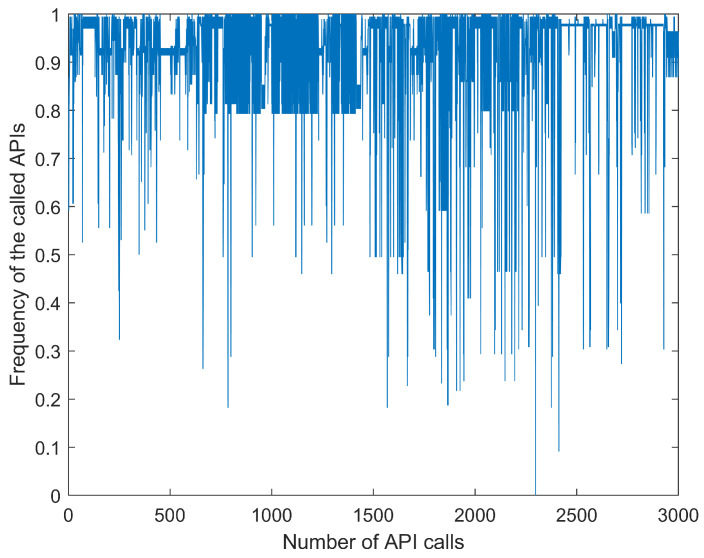
Normalized malware API call time series.

This experiment used the past seven samples to predict the current input. The size of the training input set was (7×1000); the size of the training target set was (1000×1), the size of the testing input set was (7×200), and the size of testing target set was (200×1). Figure 5 is the time series dataset obtained by replacing the API call sequence with the word frequency for which the API appeared in the whole dataset. Figure 6 shows the normalized sequence shown in Figure 5.

**Figure 7:**
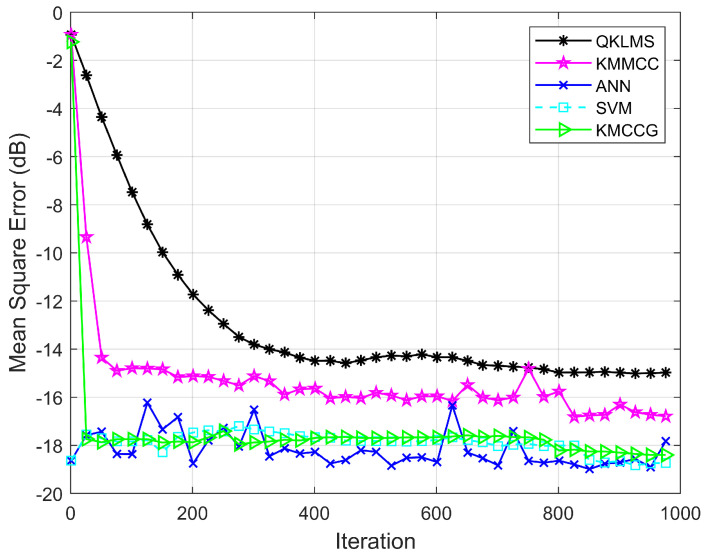
Testing MSEs of QKLMS, KMMCC, ANN (artificial neural network), SVM (support vector machine), and KMCCG for malware API call time series prediction.

In this experiment, the performance of the algorithm is verified by comparing the prediction accuracy of QKLMS, KMMCC, ANN, SVM, and KMCCG. Considering that the prediction error is an effective evaluation metric, we still adopted the transformed error (26) to evaluate the algorithm performance. Figure 7 shows the learning performance of all five algorithms, which represents the relationship between the testing MSE of the algorithm and the number of iterations. Obviously, the MSE of KMCCG is smaller than that of the classical KAF algorithms, i.e., QKLMS and KMMCC. The total training time and the average value of MSE for five runs of the experiment are summarized in Table 2. It can be found that the proposed algorithm can achieve a prediction accuracy equivalent to the popular ANN and SVM, but spent less training time. Here, the algorithm KMCCG can be successfully applied to predict malware API call sequences, which verifies the satisfactory performance of our proposed algorithm.

**Figure 8:**
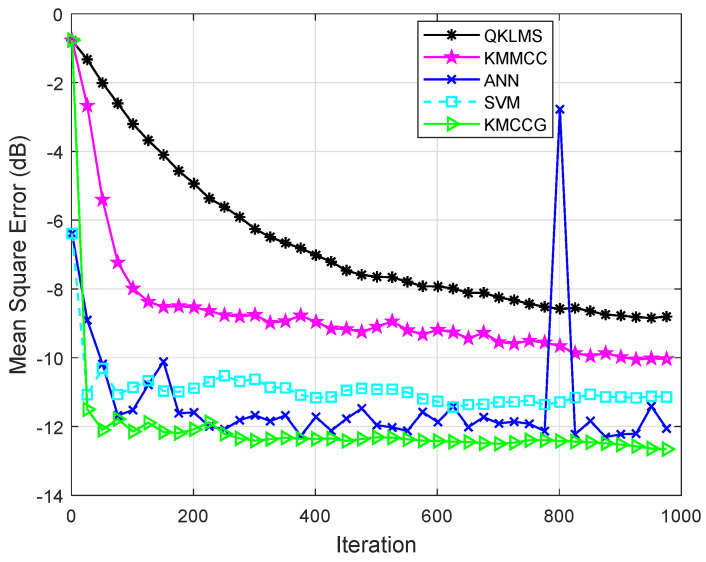
Testing MSEs of QKLMS, KMMCC, ANN, SVM, and KMCCG for malware API call time series prediction in the noise environment.

Then, the time series of malware API calls were combined with Gaussian noise to further verify the robustness of the algorithm. In the real world, noise is often not caused by a single source, but a combination of many different sources. As the number of noise sources increases, it tends to a Gaussian distribution. Here, Gaussian noise is considered in the experiment to analyze the impact of noise in the system. Figure 8 shows the performance comparison of the proposed algorithm with the other four algorithms in the Gaussian noise environment. The evaluation metric is also the MSE shown in (26). It can be seen that the algorithm KMCCG can achieve higher prediction accuracy and be more stable in the noise environment. This shows that KMCCG has satisfactory robustness.

## 5. Conclusions

Through the combination of the MCC and the algorithm KCG, a novel kernel learning algorithm, i.e., KMCCG, is proposed in this article. Specifically, in an effort to curb effectively the growing RBF network in our algorithm KMCCG, a sparsification criterion based on the angle between elements in RKHS is used to control the increase of data size in online applications, which is equivalent to the coherence criterion. The proposed algorithm achieves much faster convergence speed than the algorithm KLMS and lower computational complexity than the algorithm KRLS. The prediction results for Mackey–Glass chaotic time series and Lorentz time series showed that our algorithm achieved good performance in robustness, filtering accuracy, and computational efficiency. Furthermore, the proposed kernel learning algorithm was applied to malware prediction. The results also showed that the algorithm KMCCG not only had a short training time, but also maintained a high prediction accuracy, which further verified the satisfactory performance of KMCCG.

As a use case of our algorithm, this article only focused on the task of malware API call sequence prediction. Actually, the prediction experiment of malware API call time series is only a part of the malware detection technology, and the software cannot be directly classified as malware or a benign one by only using our method. In future work, we will combine the results of future behavior prediction with the actual detected malicious behavior as the basis of classification reference and judge whether the application belongs to malware or not. Moreover, to further extend the applications of malware prediction and detection while using the algorithm KMCCG, we will discuss some other classification tasks for malware through the evaluation of false positives.

## Figures and Tables

**Table 1:** Computational cost for dictionary update. KLMS (kernel least mean squares); KRLS (kernel recursive least squares); KCG (kernel conjugate gradient); KMCCG (kernel mixture correntropy conjugate gradient).

Algorithm	Additions	Multiplications	Divisions
KLMS	M	M	0
KRLS	$4M^{2}+4M$	$4M^{2}+4M$	1
KCG	$2M^{2}+8M$	$2M^{2}+10M$	3
KMCCG	$2M^{2}+8M+1$	$2M^{2}+10M+2$	5

**Table 2:** Computational results of QKLMS, KMMCC, ANN, SVM, and KMCCG in API call time series prediction.

Algorithm	Time (s)	MSE (dB)
QKLMS	39.03	−12.9139
KMMCC	52.46	−15.4081
ANN	841.41	−18.1565
SVM	106.21	−17.8825
KMCCG	2.24	−17.8421
